# Socio-biomedical predictors of child nutrition in India: an ecological analysis from a nationally representative Demographic and Health Survey, 2015–2016

**DOI:** 10.1186/s41043-021-00273-8

**Published:** 2022-01-03

**Authors:** Ranjan Kumar Prusty, Mohan Bairwa, Fahmina Anwar, Vijay Kumar Mishra, Kamalesh Kumar Patel, Daya Krishan Mangal

**Affiliations:** 1 Biostatistics, Indian Council of Medical Research-National Institute for Research in Reproductive Health (ICMR-NIRRH), Mumbai, India; 2 Centre for Community Medicine, All India Institute of Medical Sciences, New Delhi, New Delhi, India; 3 Medical and Scientific Affairs, Nestle S.A., Gurugram, India; 4 Public Health Foundation of India, New Delhi, India; 5 Centre for Cancer Epidemiology, Tata Memorial Centre, Mumbai, India; 6 IIHMR University, Jaipur, India

**Keywords:** Nutrition, Stunting, Wasting, Underweight, Under-five children, India

## Abstract

**Background:**

Despite significant economic growth and development, undernutrition among children remains a major public health challenge for low- and middle-income countries in the twenty-first century. In Millennium Development Goals, India committed halving the prevalence of underweight children by 2015. This study aimed to explain the geographical variation in child malnutrition level and understand the socio-biomedical predictors of child nutrition in India.

**Methods:**

We used the data from India’s National Family Health Survey 2015–2016. The survey provided estimates of stunting, wasting, and underweight at the national, state, and district level to measure nutritional status of under-five children. Level of stunting, wasting and underweight at the district level are considered as outcome variables. We have used variance inflation factor to check the multicollinearity between potential predictors of nutrition. In this study, we performed spatial analysis using ArcGIS and multiple linear regression analysis using Stata version 15.

**Results:**

Five states (Uttar Pradesh, Bihar, Madhya Pradesh, Jharkhand and Meghalaya) had very high prevalence of stunting (40% and above). High prevalence of wasting was documented in Jharkhand, Madhya Pradesh, Chhattisgarh, and Karnataka (23 to 29%). Jharkhand, Madhya Pradesh, Maharashtra, and Chhattisgarh had the highest proportion of underweight children in the country. We found that electricity and clean fuel use in the household, use of iodized salt, and level of exclusive breastfeeding had significantly negative influence on the stunting level in the districts. The use of iodized salt has similar effect on the wasting status of under-five children in the districts (b: − 0.27, *p* < 0.10). Further, underweight level had a negative association with clean fuel use for cooking (b: − 0.17, *p* < 0.01), use of iodized salt (b: − 0.36, *p* < 0.10), breastfeeding within one hour (b: − 0.18, *p* < 0.10), semisolid/solid food within 6–8 months (b: − 0.11, *p* < 0.05) and Gross Domestic Product of the districts (b: − 0.53, *p* < 0.10).

**Conclusion:**

In the study, a variety of factors including electricity and clean fuel use in the household, use of iodized salt, level of exclusive breastfeeding, breastfeeding within one hour, semisolid/solid food within 6–8 months and Gross Domestic Product of the districts have a significant association with nutritional status of children.

## Background

Since Paleolithic man emerged on Earth, the mankind has faced major shifts in dietary and physical activity patterns and body composition. Human dietary patterns and nutritional status have undergone a sequence of major shifts among characteristic states—defined as broad patterns of food use and corresponding nutrition-related diseases. Although this shift leaned toward increased obesity and noncommunicable diseases (NCDs) in recent time [24], undernutrition among children remains one of the main public health challenges of the twenty-first century, particularly in low- and middle-income countries like India [30]. The prevalence of underweight children in India is among the highest in the world, and nearly doubles that of Sub-Saharan Africa with dire consequences for morbidity, mortality, productivity and economic growth [6]. This is evident from National Family Health Survey 2015–2016 of India that almost 38 percent of the preschool children are stunted, 36 percent underweight and 21 percent wasted [15]. In this line that National Health Policy 2017 also recognized the importance of malnutrition and stated that “undernutrition restricts survival, growth and development of children. It contributes to morbidity and mortality in vulnerable populations, resulting in substantial diminution in productive capacity in adulthood and consequent reduction in the economic growth and well-being of the countries” [16]. India was committed toward halving the prevalence of underweight children by 2015 as a key indicator for achieving the Millennium Development Goal (MDG) of eradicating extreme poverty and hunger [6]. Despite the best efforts to improve child malnutrition through various national health programs including supplementary nutrition in Integrated Child Development Services (ICDS) through mid-day meal in schools [17], micronutrient initiatives, etc., the level of undernutrition remains staggering. Again, there has been wide socioeconomic disparities in health and nutritional outcomes across the geographies, religion, caste, and other socioeconomic characteristics which is debated among researchers and policy makers [10, 13, 18, 29]. The roots of this heavy toll lie in India’s inability to improve child nutrition arises from the policy environment that lacks an evidence base on socioeconomic and biomedical factors responsible for undernutrition and effectiveness of the interventions to deal with [22]. Based on this background, the present study was conducted to explain the geographical variation child malnutrition level and understand the socio-demographic and biomedical predictors of child nutrition in India.

## Methods and materials

### Ethical statement

The four waves of National Family Health Survey (NFHS) were conducted under the supervision of the International Institute for Population Sciences (IIPS), Mumbai, India, which serves as a regional institute for training and research in population studies for the Economic and Social Commission for Asia and the Pacific (ESCAP) region. Formal written consents were obtained from the respondents and ethical issues were resolved before the respondents were interviewed. This study is based on anonymous public use reports with no identifiable information about the survey participants.

### Study setting

This study used the data from India’s nationally representative survey, National Family Health Survey 2015–2016 (NFHS-4). NFHS 2015–2016 was the first of the NFHS series (NFHS 1992–1993, NFHS 1998–1999, and NFHS 2005–2006) that collected data in each of India’s 29 states and all 7 union territories. NFHS-4 also provided estimates of most indicators at the district level for all 640 districts of the country included in the 2011 Census. In NFHS-4, women aged 15–49 years and men aged 15–54 years were interviewed. This survey was conducted during January 2015 to December 2016 by 14 field agencies and gathered information from 601,509 households, 699,686 women and 103,525 men.

### Data sources

The data used for this study are the compilation of fourth round of National Family Health Survey (NFHS, 2015–2016) factsheets. The NFHS is Indian version of Demographic and Health Survey (DHS) which is a standardized survey over 90 countries with over 300 surveys worldwide. The NFHS-IV is coordinated by the International Institute for Population Sciences (IIPS) with financial support of the Ministry of Health and Family Welfare, Government of India. It provides information on important indicators of maternal and child health, fertility and mortality. NFHS-4 fieldwork for India was conducted from January 2015 to December 2016 by 14 field agencies and gathered information from 601,509 households, 699,686 women, and 103,525 men using multistage probability proportionate sampling. In this paper, we used the information collected on anthropometric measures used to measure nutritional status of under-five children of the women in 15–49 years. NFHS-4 covered all the 29 states and 7 union territories and for the first time provides information on district level (all 640 districts in India as per census 2011) estimates for many important indicators. The details of sampling procedure and data collection protocol can be found elsewhere [15].

The information from all district- and state-level factsheets was brought together in excel sheet. The relevant indicators of nutrition and other socioeconomic variables were used for the analysis. Data were filtered and cleaned before used for analysis. In addition, socioeconomic variables available in NFHS, we also used district-wise Gross Domestic Product (GDP) from data by Ministry of Statistics and Programme Implementation (MOSPI). The detailed outcome and independent variables used in the study are discussed below.

### Outcome variables

The three standard indices of physical growth identified by World Health Organization (WHO) are used as outcome variables to describe nutritional status of children. Weight for age (underweight) is an indicator of either current or past nutrition, whereas height for age (stunting) is an indicator of past nutrition. Weight for height (wasting) is a sensitive indicator of current nutrition status and the degree of wasting. These three indices are made dichotomous with Z-score below minus two standard deviation which is considered as stunted, wasted and underweight and the rest as not stunted, wasted and underweight. These indices are used to measure nutritional status of children which is used as dependent variable. These three nutritional status indicators are expressed in standard deviation unit (Z-score) from the median of the reference population.

### Independent variable

Various socioeconomic, demographic, child and maternal factors are taken into account as independent variable. Description of the independent variables is given in “Appendix 1”.

### Data cleaning and preparation

NFHS-IV, Census and GDP data have been extracted into excel files. Each state- and district-level excel files have been cleaned properly considering the missing values. After that, we have converted excel files into Stata file so that other bivariate and multivariate analyses can be performed. Each district- and state-level excel files have been cleaned properly considering the missing values.

### Statistical analysis

We used Stata (version 15) and ArcGIS (version 10.3) software to analyze data in this study. We applied similar methodology used to calculate the cutoff values for public health significance for underweight, stunting and wasting for children under 5 years of age based on the WHO child growth standards using NFHS data on malnutrition. The classification can be found elsewhere [31]. In addition to spatial analysis, we performed multiple linear regression and multivariate multiple linear regression using STATA. Multicollinearity is a high degree of correlation (linear dependency) among several independent variables. It commonly occurs when a large number of independent variables are incorporated in a regression model. In this study, we have used variance inflation factor to check the multicollinearity between independent variables. Variance inflation factor is the reciprocal of the tolerance. We have tested multicollinearity taking 22 independent variables into predictive model (see “Appendix 2”). Symptoms of multicollinearity have been observed through the VIF value. So variables having VIF > 10 have been excluded from the regression model. In this study, we have used variance inflation factor to check the multicollinearity between independent variables. Variance inflation factor is the reciprocal of the tolerance.

VIF is given by the formula

$$VIF = 1/{(}1 - R^{2} {)}$$

where (1 − *R*^2^) is the tolerance and *R*^2^, coefficient of determination.

Multivariate simple linear regression analysis was used to understand different socioeconomic and biomedical factors associated with child undernutrition level. Simple linear regression was used considering the continuous nature of the dependent variable, i.e., level of stunting, wasting and underweight at the districts. Multivariate analytical techniques represent a variety of mathematical models used to measure and quantify outcomes, taking into account important factors that can influence this relationship.

## Results

### Spatiotemporal distribution of childhood undernutrition

**Fig. 1:**
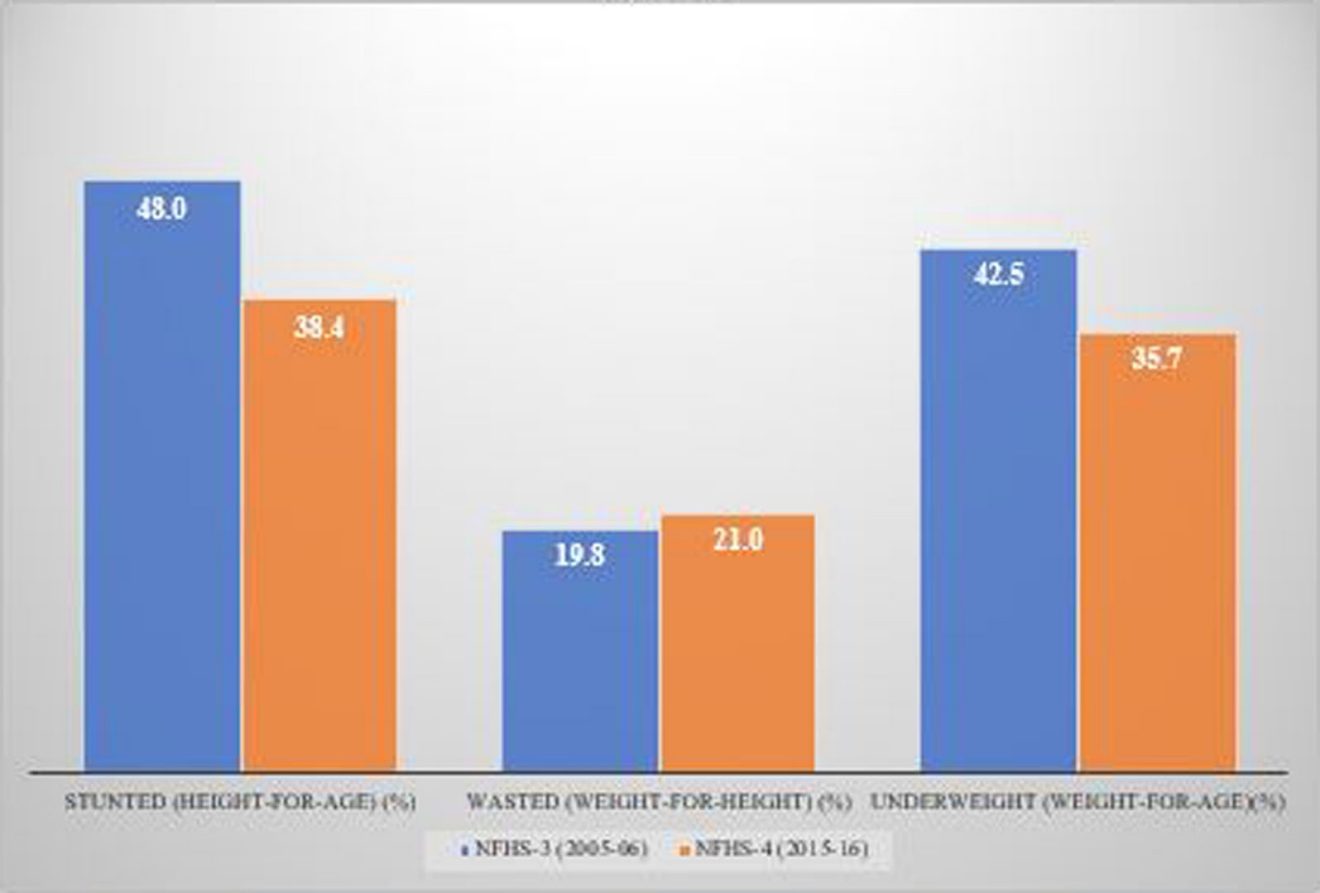
Percentage change of undernutrition indicators among under 5 year children in India, 2005–16

**Fig. 2:**
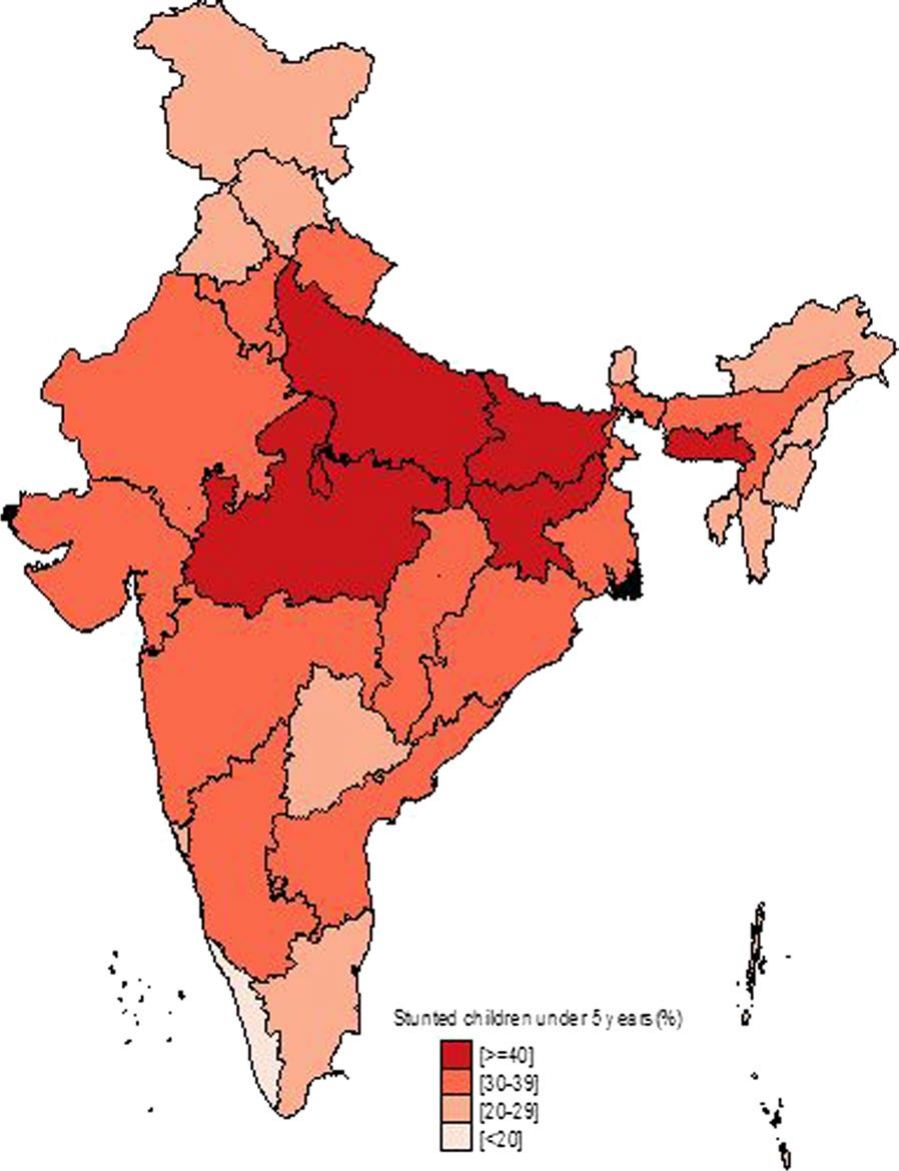
Spatial distribution of under 5 year stunted children across states of India, 2015–2016

**Fig. 3:**
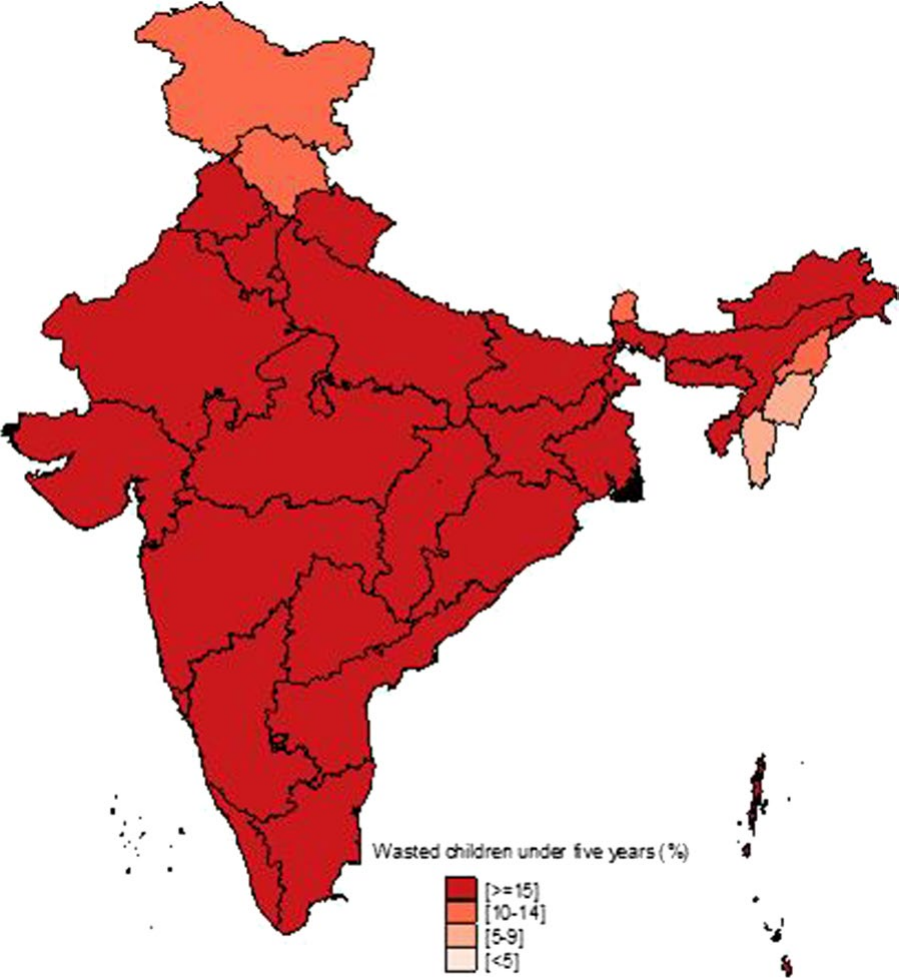
Spatial distribution of under 5 year wasted children across states of India, 2015–2016

**Fig. 4:**
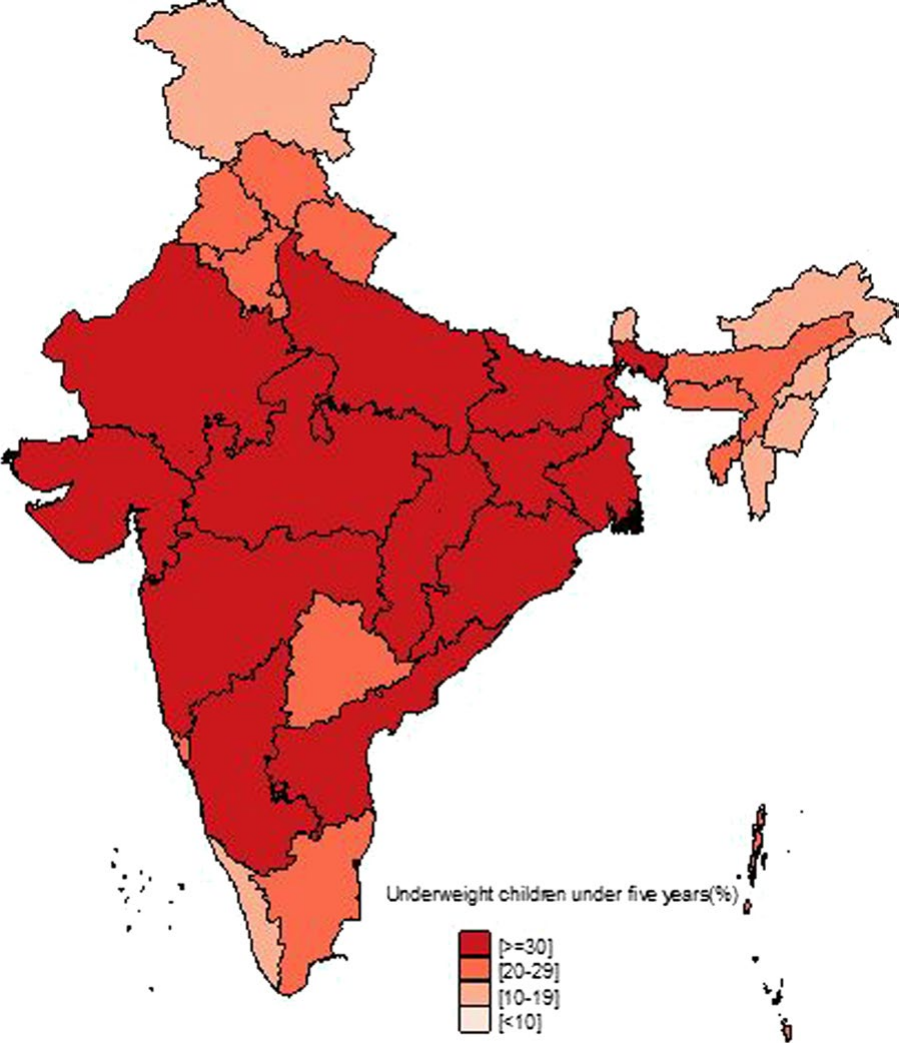
Spatial distribution of under 5 year underweight children across states of India, 2015–2016

Figure 1 shows the temporal changes in under-five child nutrition level during 2005–16. The graph shows that stunting has been declined by 10 points (48% to 38%) and underweight has gone down by 7 points (42.5% to 35.75) while wasting has gone up by 1 point (20% to 21%). Figures 2, 3 and 4 show geographical distribution of stunting, wasting and underweight under-five children across Indian states, respectively, as defined by WHO cutoff value for public health significance. Five states (Uttar Pradesh—46.3%, Bihar—48.3%, Madhya Pradesh—42.0%, Jharkhand—45.3% and Meghalaya-43.8%) had very high prevalence (≥ 40%) of stunting and 11 more states including developed states like Karnataka and Andhra Pradesh had high prevalence (30–39%) of stunting. The majority of the states are critical stage in terms of stunting according WHO classification on wasting. High proportion of wasting among the under-five years children was documented in Jharkhand (29.0%), Madhya Pradesh (25.8%), Chhattisgarh (23.1%) and Karnataka (26.1%). Among northeastern states, Arunachal Pradesh (17.3%) showed the highest proportion of stunted children. Twelve states have very high prevalence (≥ 30%) of underweight children and among them Jharkhand (47.8%), Madhya Pradesh (42.8%), Maharashtra (36.0%) and Chhattisgarh (37.7%) are the five states with the highest proportion of underweight children in the country. The detail of top 5 and bottom states in child undernutrition is shown in Table 1.

**Table 1:** Top and bottom five states on child nutritional indicators in India, 2015–2016

State	Stunted (%)	Wasted (%)	Severely Wasted (%)	Underweight (%)
Top-5	Kerala (19.7) Goa (20.1) Andaman and Nicobar Islands (23.3) Daman and Diu (23.4) Puducherry (23.7)	Mizoram (6.1) Manipur (6.8) Chandigarh (10.9) Nagaland (11.2) Jammu and Kashmir (12.1)	Manipur (2.2) Mizoram (2.3) Lakshadweep (3.3) Chandigarh (3.9) Himanchal Pradesh (3.9)	Mizoram (11.9) Manipur (13.8) Sikkim (14.2) Kerala (16.1) Jammu and Kashmir (16.6)
Bottom-5	Bihar (48.3) Uttar Pradesh (46.3) Jharkhand (45.3) Meghalaya (43.8) Madhya Pradesh (42.0)	Jharkhand (29.0) Dadra and Nagar Haveli (27.6) Gujarat (26.4) Karnataka (26.1) Madhya Pradesh (25.8)	Daman and Diu (11.9) Jharkhand (11.4) Dadra and Nagar Haveli (11.4) Karnataka (10.5) Gujarat (9.5)	Jharkhand (47.8) Bihar (43.9) Madhya Pradesh (42.8) Uttar Pradesh (39.5) Gujarat (39.3)
India	38.4	21.0	7.5	35.7

**Fig. 5:**
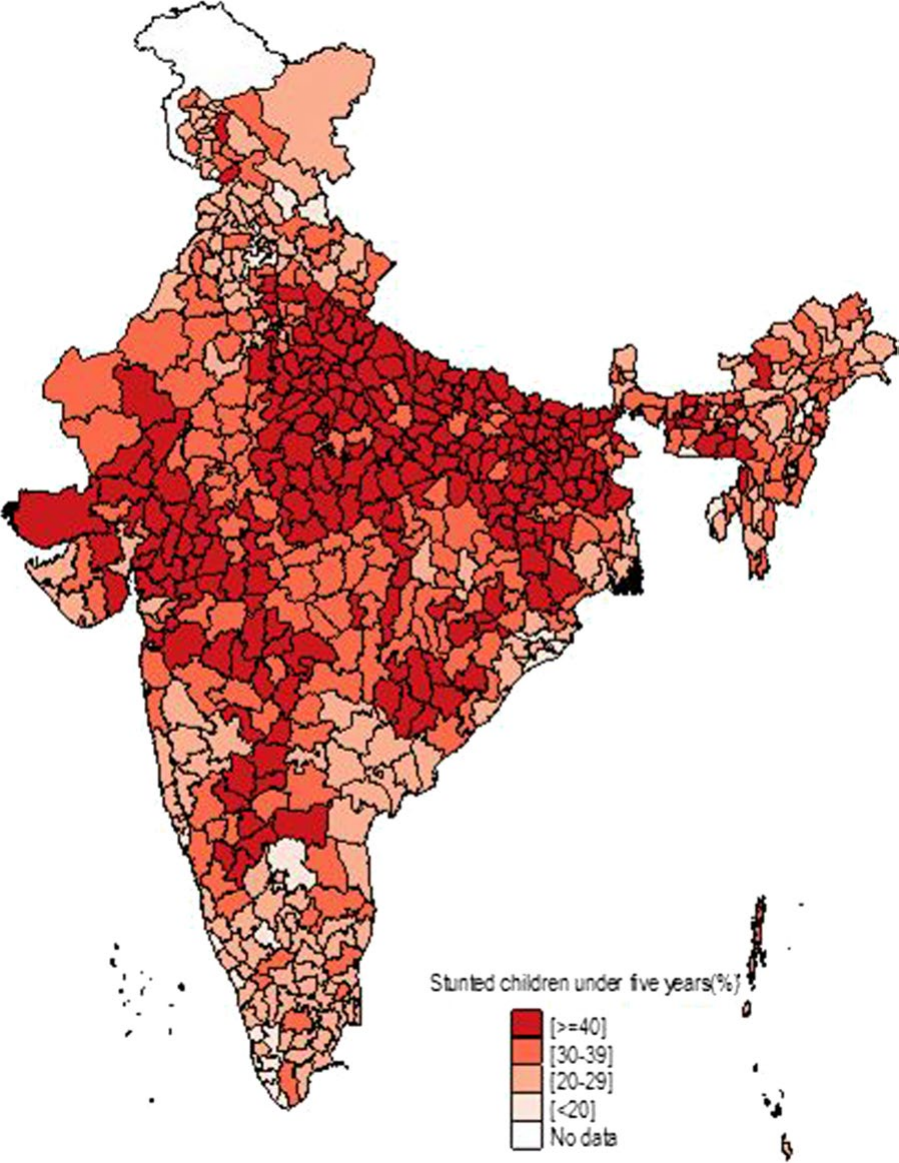
Spatial distribution of under 5 year stunted children across districts of India, 2015–2016

**Fig. 6:**
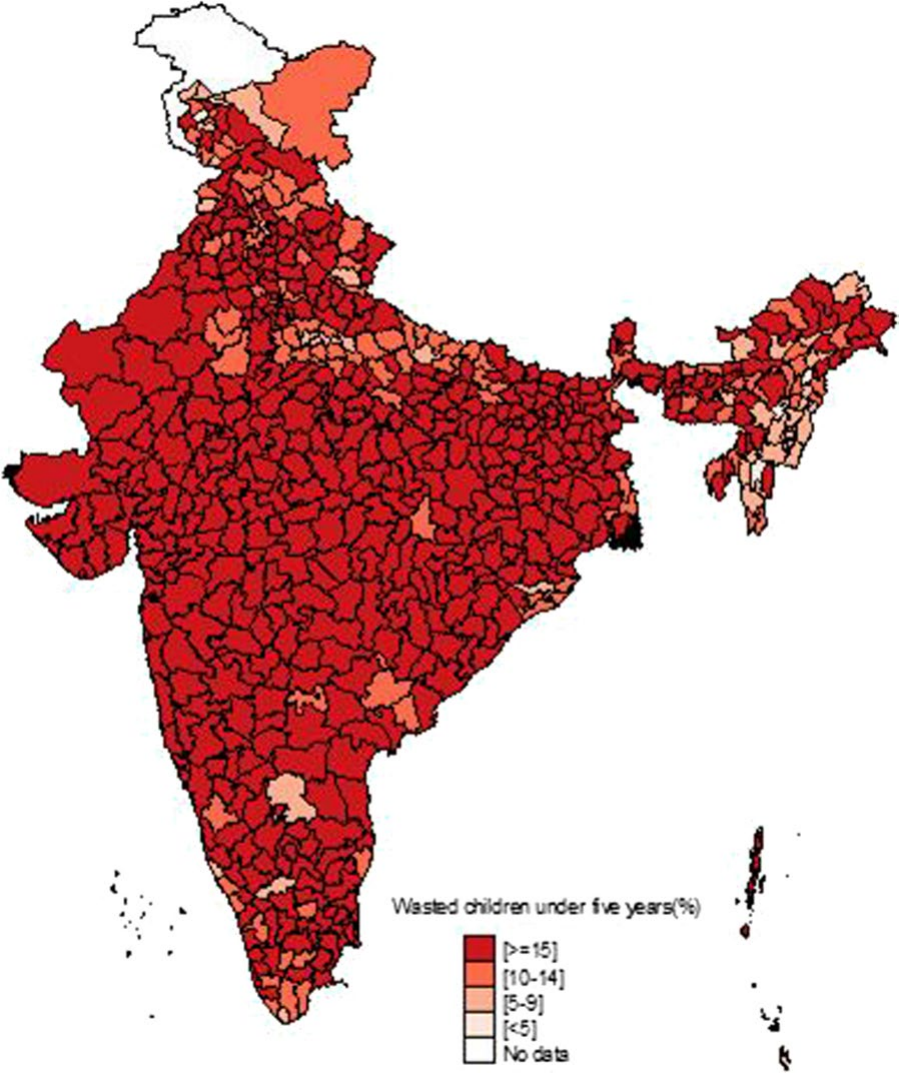
Spatial distribution of under 5 year wasted children across districts of India, 2015–2016

**Fig. 7:**
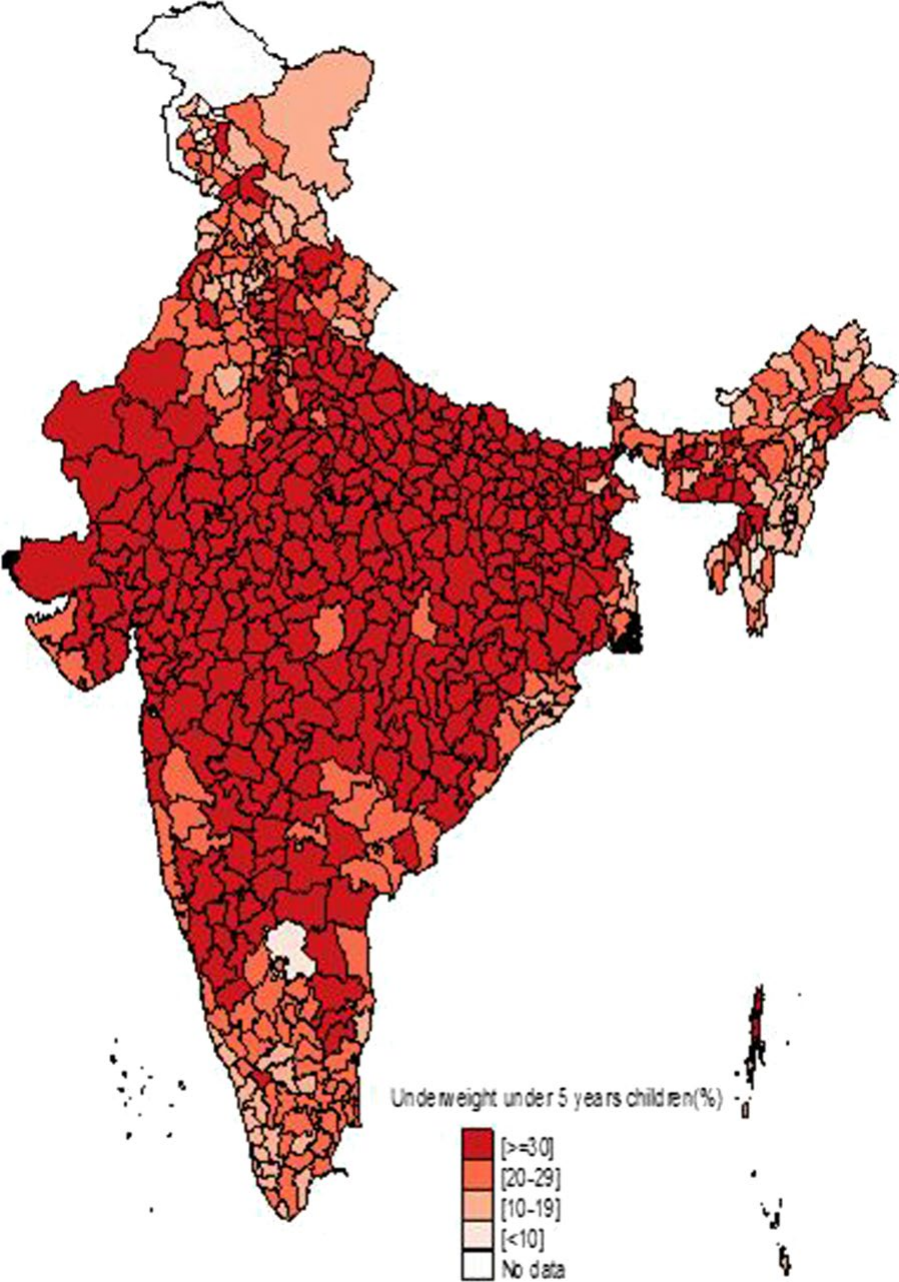
Spatial distribution of under 5 year underweight children across districts of India, 2015–16

District-level analysis showed that child undernutrition was most prevalent in districts of Uttar Pradesh, Bihar, Odisha, Rajasthan and Gujarat (Figs. 5, 6, 7). The bottom 20 districts had the highest proportion of stunting was found in the state of Uttar Pradesh (10 districts), Bihar (5 districts), Jharkhand and Karnataka (2 districts each) and Rajasthan (1 district), whereas the highest proportion of underweight children was found Jharkhand and Uttar Pradesh (4 districts each), Gujarat, Rajasthan, Bihar, and Madhya Pradesh (2 districts each), West Bengal, Maharashtra and Karnataka (1 district each) (see Table 2).

**Table 2:** Top and bottom 20 districts by child nutritional indicators in India, 2015–2016

Districts	Stunted (%)	Wasted (%)	Underweight (%)
Top-20	**Kerala-**Ernakulam (12.4), Pathanamthitta, (13.3), Kollam (14.4), Alappuzha (14.5), Idukki (15.1), Kasaragod (18.7), Kozhikode (18.0) **Odisha-**Cuttack (15.3), Puri (16.1) **Telangana-** Hyderabad (15.7) **Tamil Nadu**- Kanniyakumari (17.2) **Karnataka-**Mandya (18.6) **Goa,** South Goa (18.3) **Punjab-**Patiala (17.6), Fatehgarh Sahib (18.7) **Jammu and Kashmir**- Anantapur (18.2) **Himachal Pradesh**-Kinnaur (18.4) **Meghalaya-**South Garo Hills (16.8) **Nagaland-**Wokha (18.7) **Daman & Diu**-Daman (18.9)	**Nagaland**- Mokokchung (1.8), Peren (4.1), Phek (6.7) **Mizoram-**Aizawl (2.3), Chamrajnagar (6.1), Kolasib (6.5), Lunglei (6.1) **Assam-**Dhemaji (6.2), Dima Hasao (6.3) **Manipur-**Churachandpur (6.6), Bishnupur (6.9), Imphal West (4.7) **Puducherry**-Mahe (6.3) **Jammu and Kashmir-**Badgam (4.2), Ganderbal (4.6), Kupwara (5.1), Samba (5.2), Anantapur (5.4), Bandipore (6.2), Kargil (6.8)	**Nagaland**-Mokokchung (5.8), Peren (10.3) **Mizoram-**Aizawl (6.7), Kolasib (11.0) **Jammu and Kashmir-**Anantapur (8.2), Ganderbal (8.3), Badgam (8.0), Samba (9.0), Bandipore (9.9), Kulgam (10.2),Shupiyan (11.1) **Arunachal Pradesh**- Tawang (8.1), Anjaw (10.4) **Manipur**-Imphal West (8.4), Ukhrul (11.1), Churachandpur (11.3) **Sikkim**-East (11.2) **Himachal Pradesh**-Kullu (11.0) **Andaman and Nicobar-**Nicobar (10.5) **Kerala**-Kannur (10.5)
Bottom-20	**Uttar Pradesh-**Etawah (53.2), Maharajganj (53.3), Kheri (53.9), Budaun (55.1), Sitapur (56.4), Gonda (56.9), Siddharth Nagar (57.9), Balrampur (62.8), Shrawasti (63.5), Bahraich (65.1) **Bihar-**Sheohar (53), Vaishali (53.7), Kaimur (bhabua) (53.8), Nalanda (54.1), Sitamarhi (57.3) **Jharkhand-**Godda (54), Pashchimi Singhbhum (59.4) **Rajasthan**-Dhaulpur (54.3) **Karnataka**-Yadgir (55.5), Koppal (55.8)	**Meghalaya**-South Garo Hills (36) **Odisha**- Nabarangapur (36) **Rajasthan-**Dungarpur (37.5), Sirohi (36.6), Pratapgarh (38.2) **Gujarat-**Panch Mahals (36.3), The Dangs (43) **Maharashtra**-Garhchiroli (45.8), Nandurbar (39.8) **Uttar Pradesh**- Lalitpur (39) **Uttarakhand**- Uttarkashi (39.4), Tehri Garhwal (46.9) **Haryana-**Ambala (37.9) **Karnataka**-Gadag (43.1) **Jharkhand-** Simdega (36.7), Bokaro (36.9), Pashchimi Singhbhum (37.5), Khunti (43), Dumka (41.4), PurbiSinghbhum (40.6)	**Jharkhand-**Pashchimi Singhbhum (66.9),Khunti (53.8), Saraikela-kharsawan (52.6),Dumka (53.5) **Uttar Pradesh**- Shahjahanpur (54.3), Kaushambi (52.8), Budaun (53.6), Jaunpur (52.7) **Gujarat**-The Dangs (60.0),Narmada (53.6) **Karnataka**-Gulbarga (56.7) **West Bengal**-Puruliya (58.2) **Madhya Pradesh-**Barwani (55.0), Sheopur (55.0) **Rajasthan**-Pratapgarh (54.6), Dungarpur (53.4) **Maharashtra**- Nandurbar (55.4) **Bihar**- Arwal (54.0), Gaya (53.1) **Karnataka-** Bellary (53.3)

**Fig. 8:**
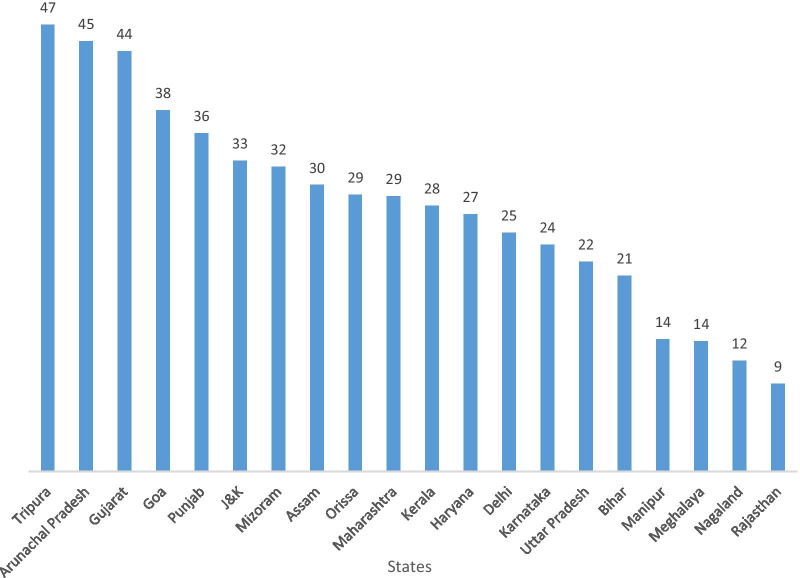
Decline in percentage points of stunted children in Indian states, 1991–2016

Comparison of NFHS-I (1991–92) and NFHS-IV (2015–2016) reveals that Tripura, Arunachal Pradesh and Gujarat had the highest proportion of decline in stunted children whereas Rajasthan and Nagaland had the lowest decline in proportion of stunting children (Fig. 8).

### Correlation between dependent and independent variables

**Fig. 9:**
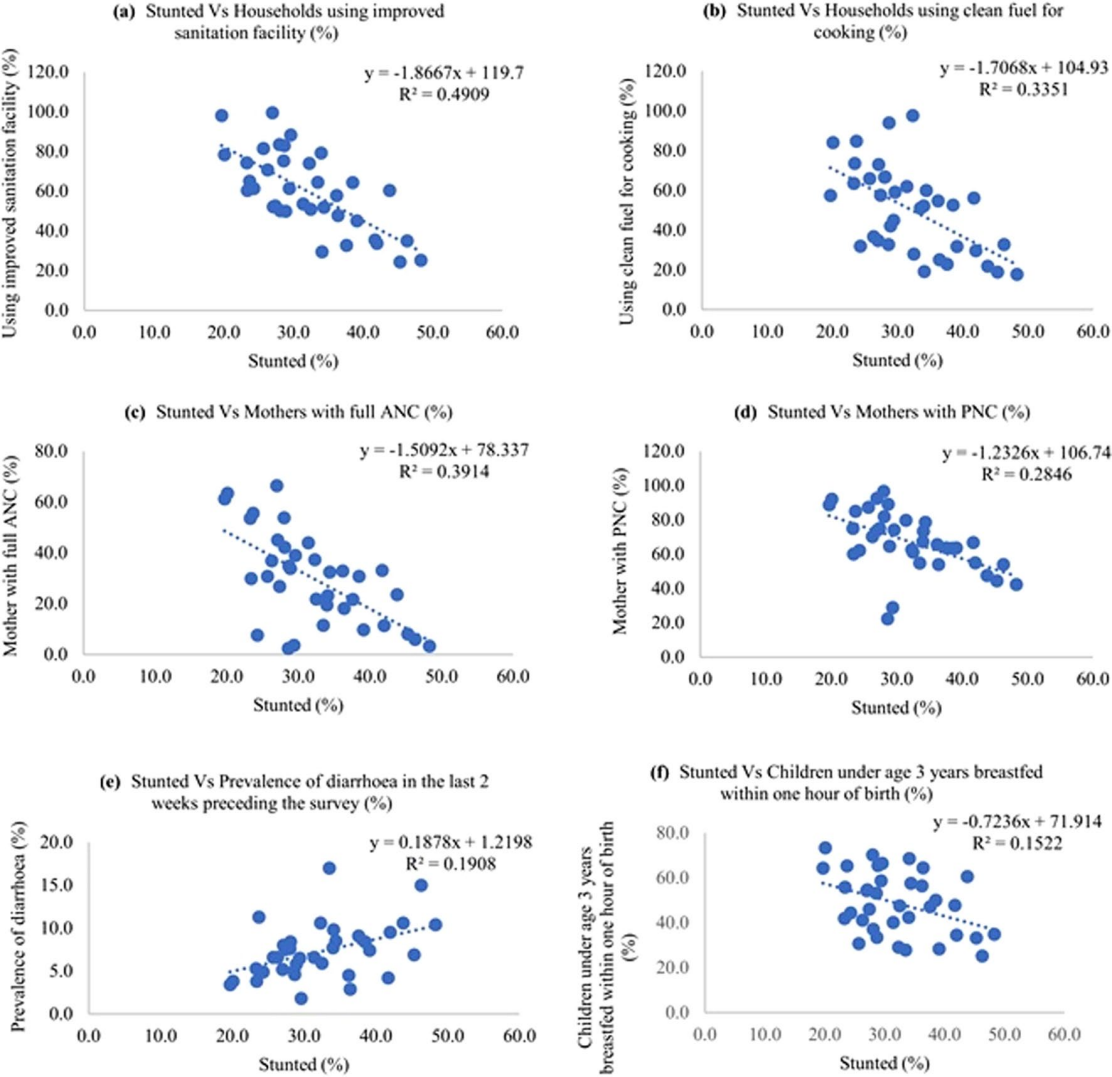
Scatter diagram showing correlation between stunted and different independent variables

**Fig. 10:**
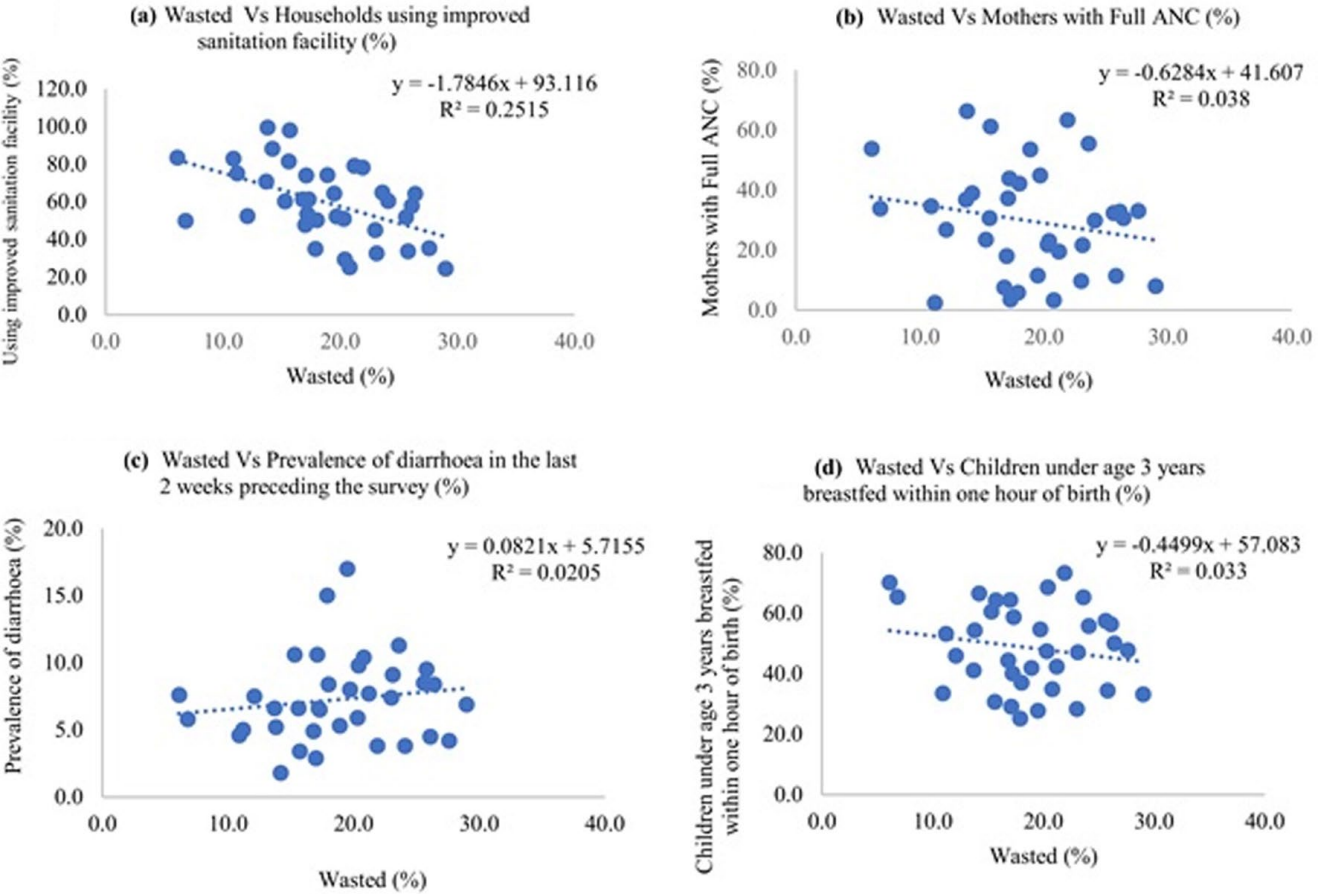
Scatter diagram showing correlation between wasted and different independent variables

**Fig. 11:**
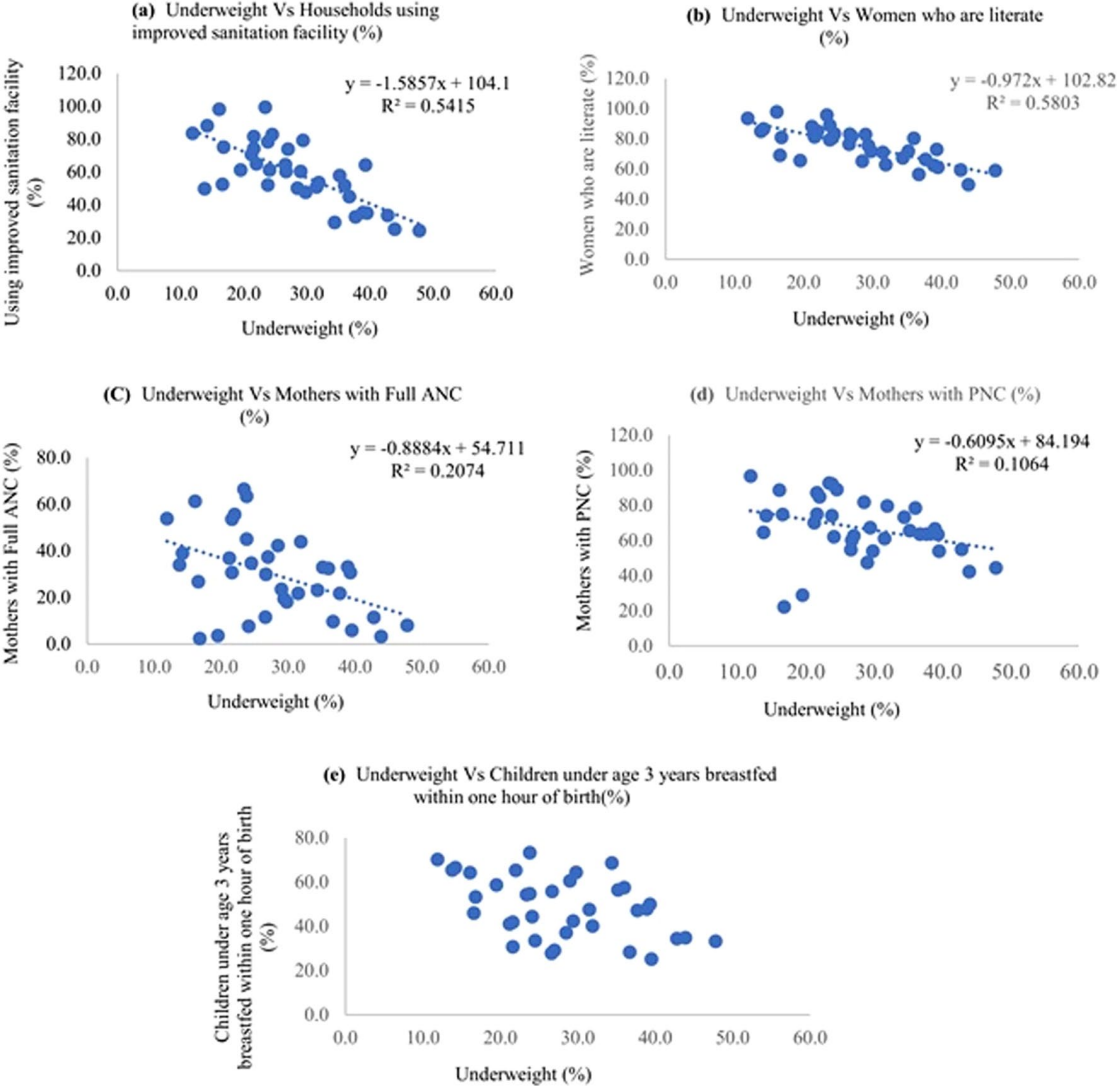
Scatter diagram showing correlation between underweight and different independent variables

The scatter diagrams showed the correlation between child nutrition level (stunting, wasting and undernutrition level) with different socioeconomic and biomedical variables (Figs. 9, 10, 11).

Stunting had an inverse correlation with better sanitation and improved cooking fuel at the household, women having access to complete antenatal care and postnatal care, and children who were exclusively breastfed for 6 months. The diarrhea incidence had a direct positive correlation with stunting among children. There was a negative correlation between wasting and complete antenatal care, exclusive breastfeeding for six months, and semisolid food initiation within 6–8 months, and children receiving adequate diet. The proportion of underweight children had negative correlation with improved sanitation level, literacy level, complete antenatal care level, postnatal care level and exclusive breastfeeding for six months.

### Multivariate analysis

Table 3 presents the results of simple linear multivariate regression analysis. Our results showed the factors which affected the level of stunting, wasting and underweight children. We found that electricity and clean fuel use in the household, use of iodized salt and level of exclusive breastfeeding had significantly negative influence on the stunting level in the districts. The use of iodized salt has similar effect (b: − 0.27, *p* < 0.10) on the wasting status of under-five children in the districts. Further, underweight level had a negative association with clean fuel use for cooking (b: − 0.17, *p* < 0.01), use of iodized salt (b: − 0.36, *p* < 0.10), breastfeeding within one hour (b: − 0.18, *p* < 0.10), semisolid/solid food within 6–8 months (b: − 0.11, *p* < 0.05) and Gross Domestic Product (GDP) of the districts (b: − 0.53, *p* < 0.10). There was a direct association between diarrhea incidence (b: − 0.32, *p* < 0.05) and underweight status of the children.

**Table 3:** Multivariate regression showing factors associated with stunted, wasted and underweight in India, 2015–2016

Variables	Coef.	*p* > *t*	[95% confidence interval]	
			Lower	Upper
*Stunted* *(Dependent Variable)*				
*Households with electricity*	− 0.25	0.05	− 0.50	0.00
HHs with an improved drinking water	− 0.11	0.19	− 0.27	0.06
*Households using clean fuel for cooking*	− 0.15	0.01	− 0.25	− 0.05
*Households using iodized salt*	− 0.31	0.03	− 0.60	− 0.03
Prevalence of diarrhea	0.32	0.29	− 0.29	0.93
Children < 3 years breastfed within one hour	− 0.12	0.11	− 0.26	0.03
*Children under age 6 months exclusive breastfed*	− 0.13	0.06	− 0.26	0.01
Children age 6–8 months receiving solid or semisolid food	− 0.01	0.83	− 0.09	0.08
GDP of district	0.20	0.47	− 0.36	0.76
SC female population	0.06	0.22	− 0.04	0.16
ST female population	0.04	0.38	− 0.06	0.15
*Wasted (dependent variable)*				
Households with electricity	0.10	0.48	− 0.18	0.38
Households with an improved drinking water	0.13	0.16	− 0.06	0.31
Households using clean fuel for cooking	− 0.07	0.23	− 0.18	0.05
*Households using iodized salt*	− 0.27	0.09	− 0.59	0.05
Prevalence of diarrhea	0.21	0.53	− 0.47	0.89
Children underage 3 years breastfed within 1 h	− 0.03	0.69	− 0.19	0.13
Children under age 6 months exclusive breastfed	0.06	0.44	− 0.09	0.21
Children age 6–8 months receiving solid or semisolid food	− 0.06	0.19	− 0.16	0.03
GDP	0.38	0.22	− 0.24	1.00
SC female population	0.06	0.25	− 0.05	0.17
ST female population	0.08	0.17	− 0.04	0.20
*Underweight (dependent variable)*				
Households with electricity	− 0.13	0.44	− 0.46	0.21
Households with an improved drinking water	0.01	0.95	− 0.21	0.22
*Households using clean fuel for cooking*	− 0.17	0.02	− 0.30	− 0.03
*Households using iodized salt*	− 0.36	0.06	− 0.74	0.01
*Incidence of diarrhea*	0.32	0.02	− 0.48	1.13
*Children underage 3 years breastfed within 1 h*	− 0.18	0.07	− 0.36	0.01
Children under age 6 months exclusive breastfed	− 0.02	0.86	− 0.19	0.16
*Children age 6–8 months receiving solid or semisolid food*	− 0.11	0.05	− 0.23	0.00
*GDP*	− 0.53	0.06	− 0.74	0.43
SC female population	0.10	0.13	− 0.03	0.22
ST female population	0.09	0.18	− 0.04	0.23

## Discussion

Despite of decline in undernutrition level in last decade, the largest chunk of global undernourished under-five children is in India with about two-fifths of children being stunted and underweight, and one-fifth were wasted in 2015–2016. The improvement in average annual decline in proportion of underweight children is evident from previous two decades. This decline has improved from 0.42 percentage point per year during 1991–2005 [21] to 0.68 percentage point (2005–2006 to 2015–2016). However, there exists a wide geographical diversity in childhood undernutrition. To achieve the global nutritional targets of World Health Assembly 2012, and SDGs targets, it is pertinent to understand spatial distribution and their sociodemographic and biomedical predictors.

We found that underweight and stunting level among under-five is highly concentrated in central and western Indian states of Rajasthan, Gujarat, Bihar, Uttar Pradesh, Jharkhand, Chhattisgarh and Madhya Pradesh. This is in coherence with national priorities of Govt of India. The twelfth five-year plan of India (2012–2017) focused on reducing the malnutrition level by half by 2017 with a special focus on the above states [23]. However, our results showed the sluggish decline during 2005–16 and is highly unlikely to achieve the targets by the year 2017. In addition, the states with better socioeconomic indicators (Non-Empowered Action Group states like Maharashtra and Karnataka) have huge proportion of wasted children. Maharashtra has substantial proportion of underweight children higher than EAG states like Odisha and Assam. The district-level analysis revealed the clustering of child undernutrition at southern districts of Odisha and Chhattisgarh, northern districts of Odisha and southern districts of Jharkhand, northern districts of Bihar and northwestern districts of West Bengal, northern districts of Uttar Pradesh and boarder districts of Uttar Pradesh, Madhya Pradesh, Rajasthan, Maharashtra and Gujarat.

These regional variations in child undernutrition are a combination of different socioeconomic variations, feeding and dietary practices, and variation in implementation of different nutritional program like Integrated Child Development Programme (ICDS) [5]. Some of the studies found an indirect relationship between access to ICDS services and undernutrition status of children in Indian states [2, 7, 19]. The utilization of ICDS services from 10 to 90% with high access to services among the states where malnutrition levels are lower and vice versa [21]. The current decline in undernutrition in the EAG states is faster because of greater budgetary allocation through National Health Mission [25]. However, the decline has been slow due to multiple factors associated to undernutrition.

The multivariate regression and scatter plot results indicated that multiple factors like use of clean fuel for cooking, use of iodized salt, mother’s education, GDP of the districts, feeding of breast milk and complementary foods are associated to different child nutrition indicators. It is found that the higher proportion of use of clean fuel is significantly associated with low stunting and underweight level of children which corroborates the findings of several other studies in India [3, 4]. Studies also showed that the indoor air pollution has significant association on child’s respiratory health which may lead to undernutrition among children [12, 28].

This was evident from previous studies that lack of use of adequately iodized salt is associated with significantly higher prevalence of malnutrition and child mortality (neonatal, infant and children aged < 5 year). However, this finding, being a cross-sectional study, does not necessarily infer the causal association between the use of iodized salt and malnutrition. Moreover, it suggested that adequately iodized salt fails to reach families who could potentially benefit the most [14, 27].

The regression result found appropriate breastfeeding practices and feeding practices are important for better nutrition among children. There are several studies indicating that inappropriate feeding practices can have greater consequences for the growth, development and survival of infants and children in Asian countries [8, 11, 26]. Foods are “gifts” of nature; human beings only cultivate the crops and rear or hunt for the animals as the case may be [20]. Nonetheless, economic growth and industrialization in recent decades led to increased production of food in India. Private distributors can use their market power to achieve high penetration of beneficial foods and micronutrients by coupling the accessibility of commercial markets with comprehensive social marketing campaigns. The private sector also represents largely untapped financial and human resources that can be mobilized in support of nutrition aims. Efforts by private–public partnerships at the international level—e.g., the Alliance for a Green Revolution in Africa (AGRA -), or the Global Alliance for Improved Nutrition (GAIN)—can be replicated at national and subnational levels and used to promote local farming, involve local commercial outlets in the distribution of nutritious food products, or support other elements of the national nutrition strategy. Involving the private sector in efforts to achieve nutrition goals carries risks—e.g., the risk of undue corporate influence on public policy, the risk of distortions in the nutrition agenda toward activities of interest to the private sector, and others. These risks are especially worrisome in countries with weak government capacity, among them many of the countries with the highest burden of undernutrition. Some guidance is available now, emphasizing the importance of developing mandatory rather than voluntary codes of conduct, and the UN Standing Committee on Nutrition has established a working group to provide guidance to countries on private sector engagement in food and nutrition programs [1].

There is no magic technological bullet to solve the problem of undernutrition. Long-term investments in the role of women—through education, economic, social and political empowerment—can be a way to deliver sustainable improvements in maternal and child nutrition, and in the health of women and children more generally. The compelling logic of this scientific evidence is that governments need national plans to scale up nutrition interventions, systems to monitor and evaluate those plans, and laws and policies to enhance the rights and status of women and children [9].

This study provided cross-sectional ecological associations which is confounded by ecological fallacy and weak evidence for causality, however, temporal ecological data, being based on nationally representative serial cross-sectional studies, may provide robust evidence.

## Conclusion

Geographic diversity in nutritional status is evident from NFHS 2015–2016 based state-level and district-level analysis. Severe undernutrition problem exists in majority of districts from central and western India. A significant number of factors found significant association with nutritional levels. Concerted efforts are required to address this variably existing public health problem through prevention model stressing on political lobbying and social policy action.

## Data Availability

All data generated or analyzed during this study are included in this published article and available at dhs.gov.in.

## Appendix 1

**Table:** Description of the independent variables for nutritional status for nutritional status of children

S. no.	Variables	Description of variables used in analysis
1	HH with electricity	House with electricity connections
2	HH with an improved drinking-water source	Household using Piped water into dwelling/yard/plot, public tap/standpipe, tube well or borehole, protected dug well, protected spring, rainwater, community RO plant
3	HH using improved sanitation facility	Households using flush to piped sewer system, flush to septic tank, flush to pit latrine, ventilated improved pit (VIP)/biogas latrine, pit latrine with slab, twin pit/composting toilet, which are not shared with any other household
4	HH using clean fuel for cooking	Family/Household using clean fuel for cooking like Electricity, LPG/natural gas, biogas
5	HH using iodized salt	Consumption of Iodized salts in different houses
6	Women literacy	Literacy level in women in each Household
7	Women literacy (More than 10 Years)	Girls/women who attended school for 10 or more years
8	Total fertility rate (children per woman)	The number of children who would be born per woman (or per 1,000 women) if she/they were to pass through the childbearing years bearing children according to a current schedule of age-specific
9	ANC in first trimester	Mothers who had antenatal check-up in the first trimester (%)
10	Mothers with at least 4 ANC visit	Mothers who had at least 4 antenatal care visits (%)
11	Full ANC	Full antenatal care is at least four antenatal visits, at least one tetanus toxoid (TT) injection and took iron folic acid tablets or syrup for 100 or more days
12	Mothers PNC	Mothers who received postnatal care from any health personnel within 2 days of delivery
13	Institutional births	Institutional delivery means giving birth to a child in a medical institution under the overall supervision of trained and competent health personnel where there are more amenities available to handle the situation and save the life of the mother and child
14	Prevalence of diarrhea in the last 2 weeks	Prevalence of diarrhea (reported) in the last 2 weeks preceding the survey
15	Children breastfed in one hour of birth	Children under age 3 years breastfed within one hour of birth
16	Exclusive breastfeeding	Children under age 6 months exclusively breastfed
17	Small child receiving solid or semisolid	Children age 6-8 months receiving solid or semisolid food and breastmilk
18	Breastfed children age 6-23 months receiving an adequate diet	Based on the youngest child living with the mother. Breastfed children receiving 4 or more food groups and a minimum meal frequency, non-breastfed children fed with a minimum of 3 Infant and Young Child Feeding Practices (fed with other milk or milk products at least twice a day, a minimum meal frequency that is receiving solid or semisolid food at least twice a day for breastfed infants 6-8 months and at least three times a day for breastfed children 9-23 months, and solid or semisolid foods from at least four food groups not including the milk or milk products food group)
19	Women BMI below normal	BMI, formerly called the Quetelet index, is a measure for indicating nutritional status in adults. It is defined as a person’s weight in kilograms divided by the square of the person’s height in meters (kg/m^2^). Women whose body mass index (BMI) is below normal (BMI < 18.5 kg/m^2^)
20	GDP at constant prices	Gross domestic product (GDP) is an inflation-adjusted measure that reflects the value of all goods and services produced by an economy in a given year, expressed in base-year prices, and is often referred to as "constant-price," "inflation-corrected" GDP or "constant dollar GDP."
21	SC population	Scheduled Castes means such castes, races or parts of groups within such castes, races as are deemed under article 341 to be Scheduled Castes for the purposes of this Constitution
22	ST population	Scheduled Castes means such races or tribes or parts of or groups within such races or tribes as are deemed under article 342 to be Scheduled Tribes for the purposes of this Constitution

## Appendix 2

**Table:** Variance Inflation Factors showing multicollinearity between potential predictors of nutrition

Variable	VIF	1/VIF
Households with electricity (%)	6.24	0.16
Households with an improved drinking water source (%)	6.93	0.14
Households using improved sanitation facility (%)	19.78	0.05
Households using clean fuel for cooking (%)	8.21	0.12
Households using iodized salt (%)	4.28	0.23
Women who are literate (%)	34.21	0.03
Women with 10 or more years of schooling (%)	19.26	0.05
Total fertility rate (children per woman)	15.65	0.06
Mothers who had antenatal check-up in the first trimester (%)	24.57	0.04
Mothers who had at least 4 antenatal care visits (%)	18.75	0.05
Mothers with PNC	13.71	0.07
Institutional births (%)	12.09	0.08
Prevalence of diarrhea (reported) in the last 2 weeks preceding the survey (%)	3.52	0.28
Children under age 3 years breastfed within 1 h of birth (%)	4.04	0.25
Children under age 6 months exclusively breastfed (%)	2.99	0.33
Children age 6–8 months receiving solid or semisolid food and breastmilk (%)	8.54	0.12
Breastfeeding children age 6–23 months receiving an adequate diet (%)	10.60	0.09
Women whose body mass index (BMI) is below normal (BMI < 18.5 kg/m^2^)14 (%)	13.70	0.07
States gross domestic product (constant price)	2.30	0.43
SC population	3.16	0.32
ST population	2.92	0.34
Mean VIF	11.88	
